# DFP-Induced Status Epilepticus Severity in Mixed-Sex Cohorts of Adult Rats Housed in the Same Room: Behavioral and EEG Comparisons

**DOI:** 10.3389/fcell.2022.895092

**Published:** 2022-05-10

**Authors:** Nikhil S. Rao, Christina Meyer, Suraj S. Vasanthi, Nyzil Massey, Manikandan Samidurai, Meghan Gage, Marson Putra, Aida N. Almanza, Logan Wachter, Thimmasettappa Thippeswamy

**Affiliations:** Department of Biomedical Sciences, College of Veterinary Medicine, Iowa State University, Ames, IA, United States

**Keywords:** sex as a biological variable, midazolam, telemetry, organophoshate, status epilepticus

## Abstract

Sex is a biological variable in experimental models. In our previous diisopropylfluorophosphate (DFP) studies, female rats required a higher dose of DFP to achieve a somewhat similar severity of status epilepticus (SE) as males. In those studies, male and female rats were bought separately from the same vendor, housed in different rooms, and the DFP used was from different batches. We had also shown that surgery for epidural electrodes implantation reduces the threshold for SE. Our recent study in the soman (GD) model using a mixed-sex cohort of rats housed individually but in the same room showed that females achieved significantly higher SE severity than males for the same dose of GD. In this study, we demonstrate that housing the mixed-sex cohorts in the same room and treating them with DFP (4 mg/kg, s.c.) from the same pool, though from different batches, yielded reproducible SE severity in both sexes and both telemetry (surgery) and non-telemetry (non-surgery) groups. We conducted experiments in four mixed-sex cohorts of adult Sprague-Dawley rats. In females, the surgery for implanting the telemetry devices reduced the latency to convulsive seizure (CS) and increased SE severity compared to non-telemetry females. However, there were no sex differences in latency or SE severity within telemetry or non-telemetry groups. Once animals reached CS stage ≥3, they remained in CS stage in both sexes until midazolam was administered. Midazolam (3 mg/kg, i.m.) treatment 1-one-hour post-DFP significantly reduced epileptiform spikes in both sexes. The mortality was only 2% in 24 h. Irrespective of sex or stage of estrous cycle or surgery, the animals had continuous convulsive SE for ∼40 min. In telemetry rats, electrographic changes correlated with behavioral seizures. However, there was a significant difference in SE severity and the latency between directly-observed behavioral CS and EEG-based CS quantification in both sexes. Overall, these results suggest that housing both sexes in the same room and treating with DFP in a mixed-sex cohort from the same pool of reagents will minimize variability in SE severity. Such rigorous experiments will yield better outcomes while testing disease-modifying agents in epilepsy models.

## Introduction

Developing experimental models that closely mimic human diseases has been challenging. Until recently, most of the experiments were done on males (Beery and Zucker, 2011). This can be a problem in translational research if a drug becomes ineffective in females or causes undesirable side effects (Franconi et al., 2007; Holdcroft, 2007; Tingen et al., 2010). Given this, the National Institute of Health has mandated the inclusion of sex as a biological variable in animal models (Clayton and Collins, 2014). Although initially, researchers perceived it as a burden to include both males and females in experimental design, its practical relevance persuaded the research community to accept and implement it in all relevant experiments.

Inclusion of females in animal models of seizures or acquired epilepsy requires meticulous planning to minimize confounding variables. Several previous elegant studies have reported variable responses to chemoconvulsants-induced status epilepticus (SE) in female rodent models (Tan and Tan, 2001; Scharfman and MacLusky, 2014; Smith et al., 2015). This is primarily due to the impact of the estrous cycle stages at the time of exposure to chemoconvulsants. Furthermore, some strains of mice or rats are resistant, while others are susceptible to the same dose of chemoconvulsant (Kosobud and Crabbe, 1990; Schauwecker and Steward, 1997; McLin and Steward, 2006). Other studies have also reported the source of the animals, housing conditions (single or grouped), age, and handling stress can contribute to variability in seizure response (Kelly, 2010; Novakova et al., 2013; Manouze et al., 2019).

Initial SE severity is a critical variable in all translational experimental models that involve testing disease modifiers after the induction of SE (Gage et al., 2021a). Achieving consistency in SE severity reduces sample size, cost, and time. In a kainic acid (KA) model, we demonstrated that repeated low doses of KA in both rat and mouse models produce reliable consistency in SE severity (Tse et al., 2014; Puttachary et al., 2016a, 2016b; Sharma et al., 2018b, 2021). This dosing modification reduced the disadvantages of a single dose of KA, such as inconsistent SE response, high mortality, and wastage of animals (Tse et al., 2014). The choice of chemoconvulsant depends on the objectives of the experiments. For instance, in organophosphate nerve agent (OPNA) toxicity, victims are exposed to a single acute dose. Thus, a repeated low-dose model is not appropriate. In the diisopropylfluorophosphate (DFP, an OPNA) rat model, a single dose of DFP (4 mg/kg) in telemetry rats yielded varying SE severity and high mortality in males, which prompted testing a lower dose of DFP in subsequent experiments (Gage et al., 2020; Putra et al., 2020a, 2020b). Our initial studies suggested that females required a higher dose of DFP to achieve the same SE response as males when the experiments were done independently (Gage et al., 2020). In subsequent studies, we challenged females with 5 mg/kg DFP rather than 4 mg/kg. We found no significant difference in initial SE severity in females treated with 5 mg/kg compared to males treated with 4 mg/kg (Gage et al., 2021a; Gage et al., 2022a). Interestingly, females had less variable SE severity at 5 mg/kg than males at 4 mg/kg. It is important to note that, though the animals were bought from the source, our previous experiments in males and females were conducted at different times with different batches of DFP (all from Sigma) due to logistic issues. Furthermore, in our previous DFP experiments, the males and females were housed in separate rooms.

Recently, in a soman (GD, an OPNA) model, we surprisingly found that the female rats without telemetry device implantation had significantly higher SE-severity than the males at the same dose of soman (132 μg/kg, 1.2 LD_50_) (Gage et al., 2022b). In that study, both males and females were housed in the same room, but in separate cages, for several days before soman exposure. We thought co-housing may have synchronized the estrous and yielded relatively consistent higher SE severity to soman challenge. However, the vaginal cytology did not provide evidence for estrous cycle synchronization at the time of soman exposure, and there was no correlation between the stages of the estrous cycle and SE severity (Gage et al., 2022b). We speculated that handling stress during the vaginal lavage might have reduced the seizure threshold in females in soman studies. In this DFP study, based on our recent soman results in the mixed-sex cohort, we exposed the mixed-sex cohort of rats housed in the same room to 4 mg/kg of DFP. In this study, we used both male and female rats implanted with or without telemetry devices. In females, we did not perform vaginal cytology to rule out the impact of handing stress on SE severity outcome in this study. The findings from these rigorous experiments are discussed here.

## Methods

### Animals, Care, and Ethics

We used 96 adult Sprague Dawley rats (49 females and 47 males) in this study. The mixed-sex cohort of animals was purchased from Charles River (Wilmington, MA, United States ). All animals were challenged with DFP. The procedures were approved by the Institutional Care and Use Committee (IACUC protocols: 21-109 and 21-110) at Iowa State University (ISU). Animals were single housed (male and females in the same room but in separate cages) with 12-hour light and dark cycles and given *ab libitum* access to food and water. At the end of each experiment, all animals were euthanized with pentobarbital sodium (100 mg/kg, i.p.) as per the American Veterinary Medical Associations Guidelines for the Euthanasia of Animals. The euthanasia solution was purchased from the Lloyd Veterinary Medical Center Hospital Pharmacy, Ames, Iowa.

### Chemicals

We prepared DFP (Sigma-Aldrich, purity, 97.8% by GC-MS) fresh in cold phosphate-buffered saline (PBS) before administration. Atropine sulfate (ATS, 99.9% pure by LC-MS, Tokyo Chemical Industry (TCI), United States ) and 2-pralidoxime (2-PAM, 99.4% pure by LC-MS, Sigma) were prepared fresh in saline. Midazolam (MDZ) was purchased from the ISU Lloyd Veterinary Medical Center Hospital Pharmacy. Before administering the drugs to animals, their identity and purity were authenticated by LC/GC-MS at the Metabolomics Laboratory, Iowa State University, Ames, IA, United States.

### Surgical Procedure for Telemetry Devices Implantation

We implanted telemetry devices in 35 rats (18 females and 17 males) about 10–12 days prior to the DFP exposure. We used the CTA-F40 PhysioTel™ devices (Data Science International (DSI), Minneapolis, MN, United States ) to acquire integrated video-EEG. The device consists of bipotential electrodes for single-channel recording. As described in our previous publications, we implanted the electrodes bilaterally, one on each side on the surface of the cortex over the dura mater, and the device was placed in a subcutaneous pouch (Puttachary et al., 2016a; Putra et al., 2020a, 2020b; Sharma et al., 2021; Gage et al., 2022b). Before surgery, the animals were administered an analgesic, buprenorphine (0.3 mg/kg, s.c.). Anesthesia was induced by 3.0% isoflurane (flow rate at 1 L/min O_2_) and maintained at 1.0–1.5% during surgery. We used Somnoflo anesthetic equipment (Kent Scientific, CT, United States ). An artificial tears ointment was applied to prevent the drying of the eyes. After implanting the electrodes and the device, the incision was closed with sterile surgical clips. The standard postoperative care was given. Vetropolycin, a triple antibiotic ointment applied to the surgical site, Baytril (5 mg/kg, s.c., Bayer Pharma, PA, United States), and 1 ml of normal dextrose saline were administered. After recovery, the animals were individually caged and placed on PhysioTel receivers (RPC-1) connected to the Data Exchange Matrix 2.0 (DSI) for acquiring video-encephalography (vEEG) using the Ponemah Acquisition software. The baseline EEG was recorded to cover both day and night cycles to evaluate the impact of surgery on brain electrical activity. The telemetry devices have sensors to record body temperature and locomotor activity.

### Status Epilepticus Induction With DFP and Seizures Scoring

All animals were randomized irrespective of sex and coded before exposure to DFP. We exposed the rats to DFP in four batches. The first three batches included non-telemetry animals (i.e., the animals without surgery). The fourth batch had telemetry animals (i.e., the animals had surgery for telemetry devices implantation). Each cohort had an equal number of males and females (±1 animal in some batches). The number of animals in each batch is given in Table 1. Irrespective of the batches or sex or telemetry or non-telemetry, all animals were challenged with 4 mg/kg DFP (s.c.) followed immediately (<1 min) by 2 mg/kg ATS (i.m.) and 25 mg/kg 2-PAM (i.m.) to reduce the peripheral effects of AChE inhibition. Most of the animals developed behavioral seizures in <10 min after DFP injection, and 1 h later, midazolam (MDZ, 3 mg/kg, i.m.) was administered to limit mortality.

**TABLE 1:** The sample size used in different cohorts of experiments. The animals that died during SE were excluded from the analysis.

Batch #	# Of females	# Of males	Non-telemetry or telemetry
I	12	12	Non-telemetry
II	12	12	Non-telemetry
III	7 (1*)	6	Non-telemetry
IV	18	17 (1*)	Telemetry
Total	49	47	*2.04% mortality during SE

In this study, behavioral SE severity is defined as the duration (in minutes) of convulsive seizures (stage 3 and above) an animal spends between DFP and MDZ treatments. Animals were directly observed and scored, by two experimenters, on each minute for the seizure stage based on a modified Racine scale (Racine, 1972) as described in our previous publications (Putra et al., 2020a; Gage et al., 2020, 2022b). In telemetry rats, in addition to the integrated vEEG acquisition, the seizures were also scored by direct observation (independent of vEEG). Stage 1 was characterized by salivation, lacrimation, urination, defecation, and mastication; Stage 2 was characterized by head nodding and tremors; Stage 3 was characterized by rearing, Straub tail, and forelimb extension; Stage 4 presented with the loss of the righting reflex and forelimb clonus, and Stage 5 included repeated rearing, falling, and circling. To measure seizure duration and severity, we calculated the number of minutes in which each animal was in a convulsive seizure (CS) (stages 3–5); stages 1 and 2 were considered non-convulsive seizures (NCS).

### Integrated Video-EEG Based Status Epilepticus, Epileptiform Spikes, and Power Spectra Quantification

The baseline EEG from a day and night cycle was used to normalize the post-DFP EEG to accurately detect stage-specific epileptiform spikes and seizures. We used the NeuroScore 3.4.0 software for offline data analysis. The default settings for artifacts such as electrical noise, exploratory behavior, and grooming were identified and excluded from epileptiform spike analysis as described previously (Tse et al., 2014; Puttachary et al., 2015, 2016a; Sharma et al., 2018a, 2018b). The baseline EEG characteristics for each animal were used to set the threshold for spike amplitude. The values were summed across groups at different time points for male and female rats. Seizures on EEG were identified using an automated seizure detection module in NeuroScore with predetermined criteria, i.e., high ampliture and high frequency spike trains lasting at least 20 s with minimum intervals of 0.05 s and maximum intervals of 1 s. NeuroScore calculated the average duration of each seizure episode and the total time spent in a seizure with these parameters. All seizure events on EEG were manually verified for behavioral CS from an integrated video and the power spectrum in NeuroScore. Electrographic seizures and spikes per minute for each animal were generated, and the data were processed for statistical analysis and graphing. We calculated the average power, each one-minute epoch, over the 1 hour between DFP and MDZ and one-hour post-MDZ. The powerbands included the delta (0.5–4 Hz), theta (4–8 Hz**),** Alpha (8–12 Hz), Sigma (12–16 Hz), Beta (16–24 Hz), and Gamma (24–80 Hz).

### Experimental Design, Statistics, and Rigor

The animals were randomized, ignoring sex and the estrous cycle stages, and the experimental groups were blinded until the data were analyzed. The normality of the data was tested with the Shapiro-Wilk test. We used GraphPad 9.0 for the statistical analysis and graphing. The specific statistical tests are outlined in the corresponding figure legends and Table 2. We had taken measures to minimize variables as in our previous studies: 1) behavioral seizure severity during SE was quantified by both direct observation and offline video analysis by at least two independent observers; 2) authentication of the identity and purity of key chemicals by LC/GC-MS; and 3) seizure events on EEG were manually verified with integrated video module for behavior and power spectrum in NeuroScore.

**TABLE 2:** Statistical tests and the values with ± SEM.

Figure 1	Non-telemetry	Telemetry	*p* value	Statistical analysis
A	5.259 ± 0.2095	4.147 ± 0.2538	0.0006***	Mann-Whitney test
B	5.433 ± 0.2612	4.111 ± 0.3605	0.0027**	Mann-Whitney test
C	5.071 ± 0.3330	4.188 ± 0.3676	0.0740	Mann-Whitney test
	Males	Females		
D	5.071 ± 0.3330	5.433 ± 0.2612	0.1875	Mann-Whitney test
E	4.188 ± 0.3676	4.111 ± 0.3605	0.9366	Mann-Whitney test
Figure 2	Non-Telemetry	Telemetry	*p* value	Statistical analysis
B	43.73 ± 1.840	50.50 ± 1.126	0.0062**	Mann-Whitney test
D	41.58 ± 2.602	51.28 ± 1.060	0.0050**	Mann-Whitney test
F	46.11 ± 2.568	49.63 ± 2.029	0.2452	Mann-Whitney test
Figure 3B	Males	Females	*p* value	Statistical analysis
	46.11 ± 2.568	41.58 ± 2.602	0.0796	Mann-Whitney test
Figure 3D	49.56 ± 2.035	51.28 ± 1.060	0.9795	Mann-Whitney test
Figure 5	Non-Telemetry	Telemetry	*p* value	Statistical analysis
B	Pre: 398.3 ± 25.47	Pre: 334.9 ± 24.42	<0.0001****	2-way ANOVA (Sidak’s multiple comparison)
	Post: 204.8 ± 43.25	Post: 121.8 ± 20.36	<0.0001****	
C	43.3 ± 1.734	35.48 ± 2.937	0.0596	Mann-Whitney test
Figure 6	Males	Females	*p* value	Statistical analysis
A	0–15 min: 3.42 ± 0.67	0–15 min: 2.43 ± 0.51	0.6841	Mixed-effects analysis (Sidak’s multiple comparison)
	16–30 min: 12.14 ± 0.96	16–30 min: 10.55 ± 0.89	0.6644	
	31–45 min: 11.32 ± 0.96	31–45 min: 10.94 ± 1.06	0.9983	
	46–60 min: 12.82 ± 0.65	46–60 min: 10.88 ± 1.10	0.4665	
B	0–15 min: 11.23 ± 0.61	0–15 min: 11.07 ± 0.64	0.9996	Mixed-effects analysis (Sidak’s multiple comparison)
	16–30 min: 13.92 ± 0.49	16–30 min: 14.29 ± 0.26	0.9510	
	31–45 min: 14.33 ± 0.25	31–45 min: 14.36 ± 0.19	>0.9999	
	46–60 min: 13.83 ± 0.11	46–60 min: 13.86 ± 0.09	0.9997	
C	Behavioral seizure	Electrographic seizure	*p* value	Statistical analysis
	Male: 12.33 ± 0.33	Male: 3.42 ± 0.67	<0.0001****	2-way ANOVA (Sidak’s multiple comparison)
	Female: 12 ± 0.36	Female: 2.43 ± 0.51	<0.0001****	
D	Male: 13.92 ± 0.49	Male: 12.14 ± 0.96	0.1679	2-way ANOVA (Sidak’s multiple comparison)
	Female: 14.29 ± 0.26	Female: 10.55 ± 0.89	0.0008***	
E	Male: 14.33 ± 0.25	Male: 12.16 ± 0.51	0.0515	2-way ANOVA (Sidak’s multiple comparison)
	Female: 14.36 ± 0.19	Female: 10.94 ± 1.06	0.0006***	
F	Male: 13.83 ± 0.11	Male: 12.82 ± 0.65	0.5277	2-way ANOVA (Sidak’s multiple comparison)
	Female: 13.86 ± 0.09	Female: 10.88 ± 1.10	0.0042**	
Figure 7	Males	Females	*p* value	Statistical analysis
F (α)	19.25 ± 3.70	18.09 ± 2.36	0.8771	Mann-Whitney test
F (β)	16.55 ± 3.60	14.38 ± 2.17	0.6048	Unpaired *t*-test
F (γ)	18.77 ± 3.29	19.04 ± 2.22	0.9465	Unpaired *t*-test
F (δ)	82.52 ± 25.47	136.6 ± 35.05	0.1546	Mann-Whitney test
F (θ)	32.53 ± 8.65	41.80 ± 8.02	0.1279	Mann-Whitney test
F (σ)	12.92 ± 2.53	11.04 ± 1.69	0.7838	Mann-Whitney test
G (α)	6.12 ± 1.10	5.07 ± 1.16	0.2972	Mann-Whitney test
G (β)	5.34 ± 1.04	3.92 ± 0.97	0.1932	Mann-Whitney test
G (γ)	8.05 ± 1.83	5.28 ± 1.06	0.2520	Mann-Whitney test
G (δ)	33.11 ± 8.33	81.06 ± 27.18	0.1598	Mann-Whitney test
G (θ)	13.01 ± 2.90	14.61 ± 4.06	0.9798	Mann-Whitney test
G (σ)	4.22 ± 0.80	3.05 ± 0.72	0.2116	Mann-Whitney test
Figure 8	Males	Females	*p* value	Statistical analysis
A	EEG: 10.54 ± 0.9649	EEG: 10.64 ± 0.7457	<0.0001****	2-way ANOVA (Sidak’s multiple comparison)
	Behavior: 3.692 ± 0.3985	Behavior: 4 ± 0.3780	<0.0001****	
B	6.846 ± 0.8537	6.643 ± 0.5800	0.8364	Mann-Whitney test
C	EEG: 40.29 ± 3.412	EEG: 35.48 ± 2.937	0.0106*	2-way ANOVA (Sidak’s multiple comparison)
	Behavior: 50.85 ± 2.142	Behavior: 52.5 ± 0.9478	<0.0001****	

## Results

### Latency to Behavioral CS Onset: Non-Telemetry Versus Telemetry and Sex Differences

**FIGURE 1:**
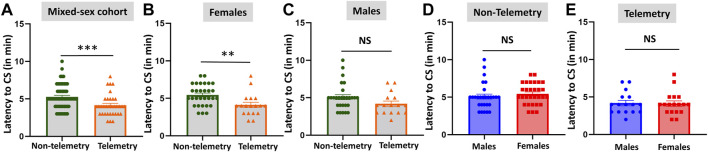
Latency to the onset of convulsive seizures (CS) in non-telemetry and telemetry animals, and sex differences. Non-telemetry and telemetry mixed-sex cohorts **(A)**, females **(B)**, and males **(C)**. A significant reduction in latency was observed in the telemetry group **(A)** driven by females **(B)**. There were no significant sex differences within the non-telemetry **(D)** or telemetry **(E)** group. Mann-Whitney test [**(A)** *n* = 34–58; **(B)** *n* = 18–30; **(C)** *n* = 16–28; **(D)** *n* = 28–30; **(E)** *n* = 16–18]. **p = 0.0027, ***p = 0.0006. NS, non-significant.

The telemetry device implanted animals in the mixed-sex cohort had significantly lower latency to the onset of the first behavioral CS compared to the non-telemetry animals (*p* = 0.0006; Figure 1A). Further analysis of sex differences within telemetry and non-telemetry groups revealed that the difference in latency was only in females (*p* = 0.0027; Figure 1B). Though there was a reduction in latency in the telemetry males, the difference was not significant (Figure 1C). Based on direct observation by experimenters, both males and females had an average of 5 min latency in the non-telemetry group and 4 min in the telemetry group (Figures 1D,E). One out of 96 rats did not show CS, and it was excluded from the study. The exact values, statistical tests, and *p*-values are given in Table 2.

### Behavioral Status Epilepticus Severity in Mixed-Sex Cohorts: Non-Telemetry Versus Telemetry and Sex Differences

**FIGURE 2:**
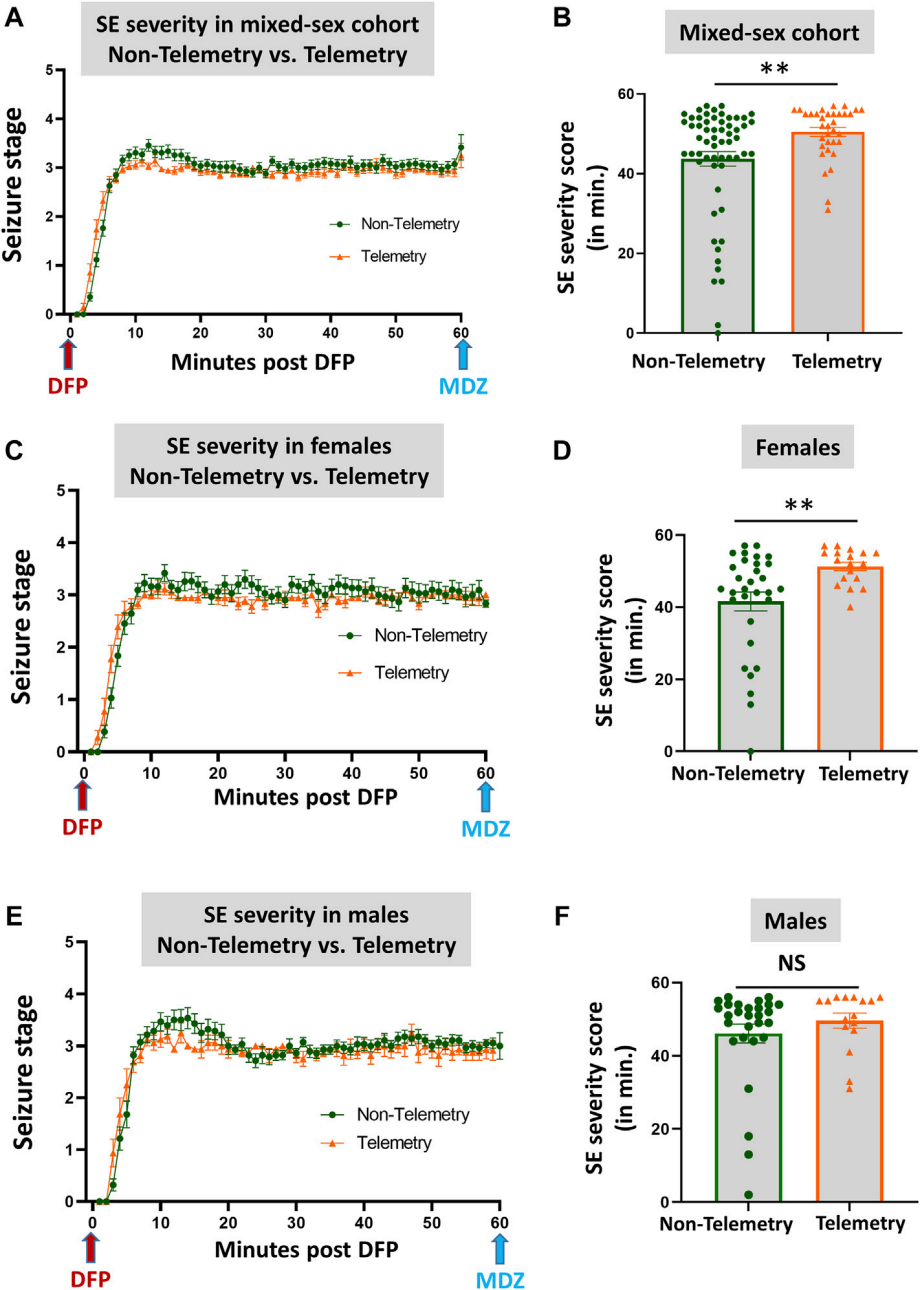
SE severity comparison between non-telemetry and telemetry animals as a mixed-sex cohort **(A,B)**. SE severity was significantly increased in the telemetry group **(B)** and females **(D)** compared to the non-telemetry group. In males, no differences were observed between the telemetry and non-telemetry groups in the SE severity **(F)**. Mann-Whitney test [**(A,B)** *n* = 34–59; **p = 0.0062; **(C,D)** *n* = 18–31, **p = 0.005; **(E,F)** *n* = 16–28]. NS, non-significant.

**FIGURE 3:**
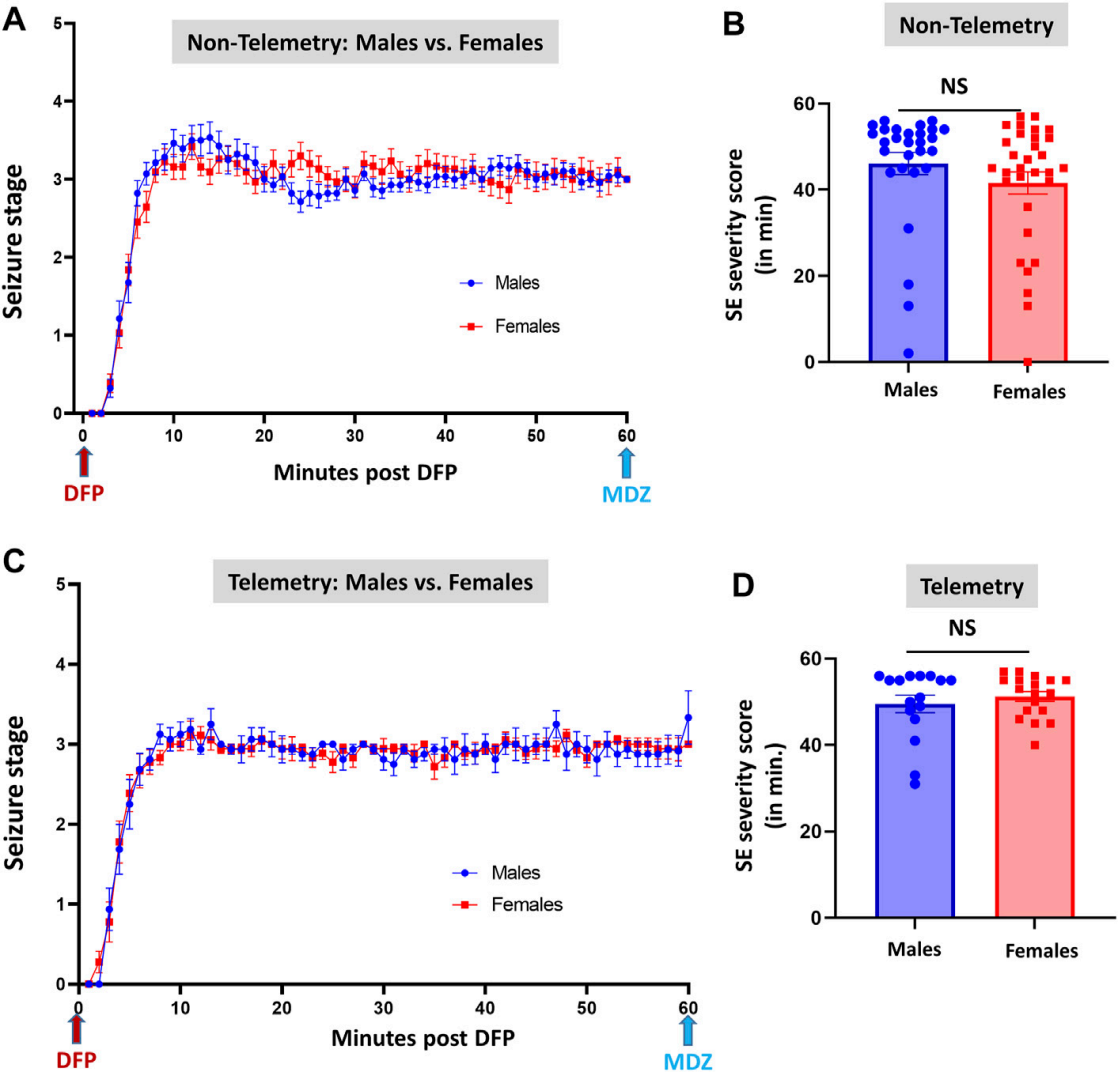
Comparison of SE severity between male and female animals in non-telemetry **(A,B)** and telemetry **(C,D)** groups. No significant differences were observed between sexes. NS, non-significant.

Based on direct observation of behavioral CS in mixed-sex cohorts, telemetry animals had significantly more severe SE (duration of CS with stage ≥3) than non-telemetry animals (*p* = 0.0062; Figures 2A,B). The telemetry animals had 50.50 ± 1.126 min of CS, and non-telemetry animals seized for 43.73 ± 1.840 min (Table 2). Further analysis of sex differences in telemetry and non-telemetry groups revealed a significant difference in SE severity in females (*p* = 0.005) but not in males (Figures 2C–F). There were no significant differences when sex was compared within the non-telemetry and telemetry groups (Figure 3). The statistical tests and *p*-values for each comparison are included in Table 2.

### EEG-Based Status Epilepticus Severity, Epileptiform Spikes, Power, Midazolam Effect, and Sex Differences

**FIGURE 4:**
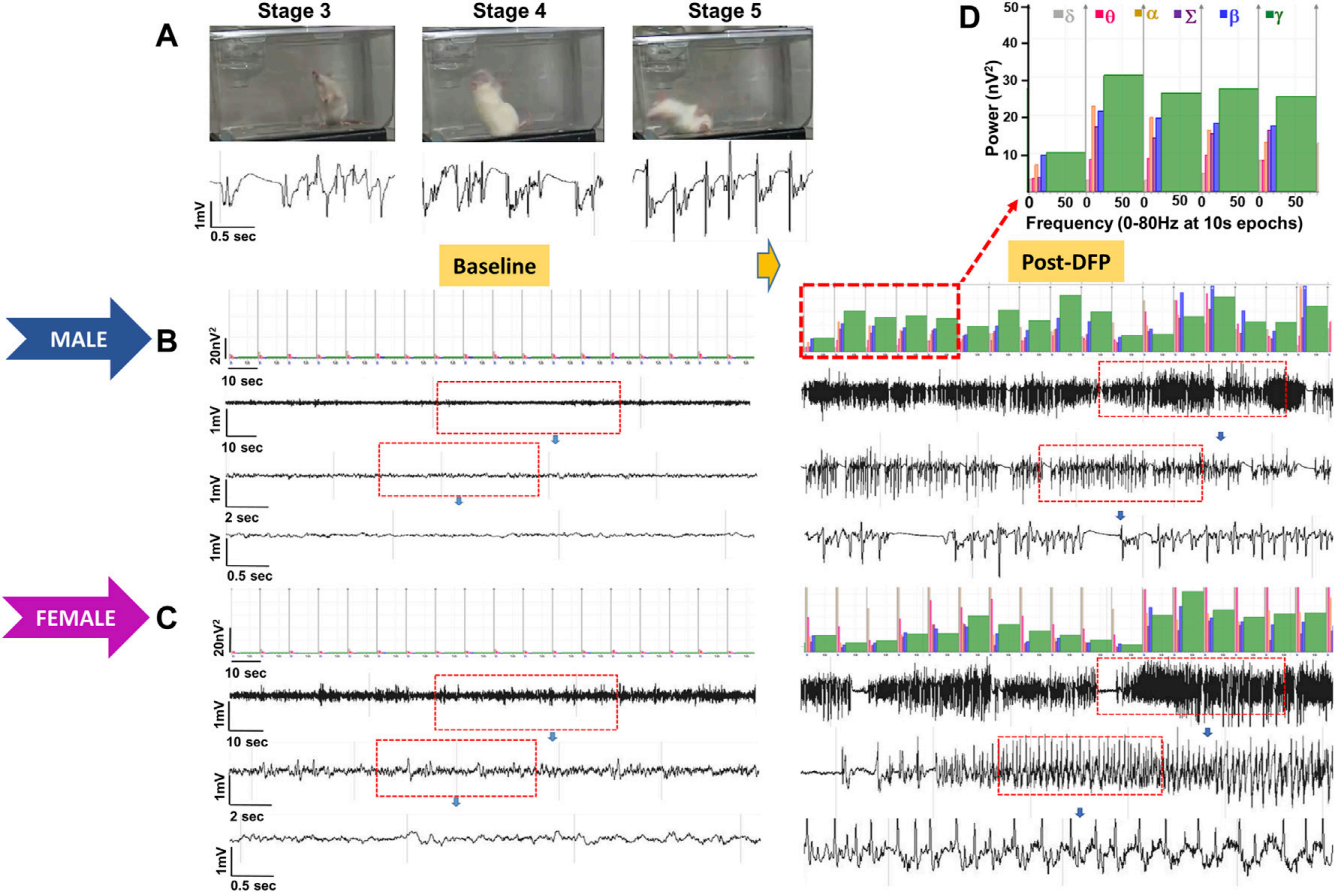
Representative convulsive seizure stages 3–5 and the corresponding stage-specific spike characteristics are presented **(A)**. Representative baseline and post-DFP EEG traces and the corresponding power spectrum from the male **(B)** and female **(C)** are shown.

**FIGURE 5:**
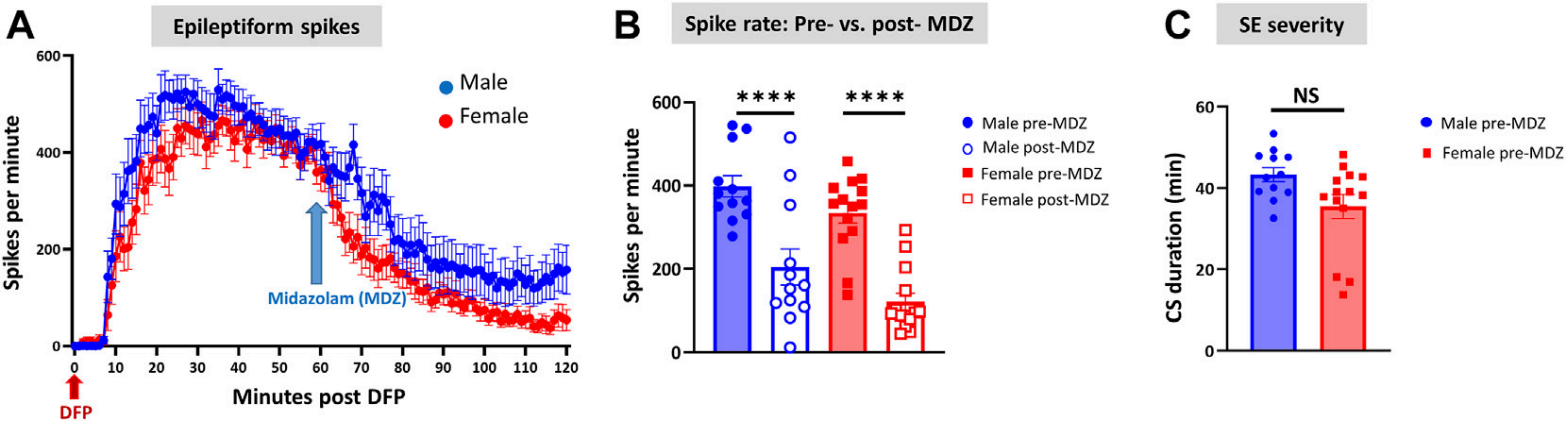
Epileptiform spikes, latency, and SE severity quantification. Epileptiform spike rate and electrographic seizures were compared between post-DFP (i.e., pre-MDZ) and post-MDZ in males and females. The spike rate was significantly reduced in both sexes after MDZ **(A,B)**. There was no difference between males and females in the electrographic seizures duration between DFP and MDZ injections **(C)**. 2-way ANOVA [**(B)** *n* = 12–14], Mann-Whitney test [**(C)** *n* = 12–14]. NS, non-significant ****p < 0.0001.

**FIGURE 6:**
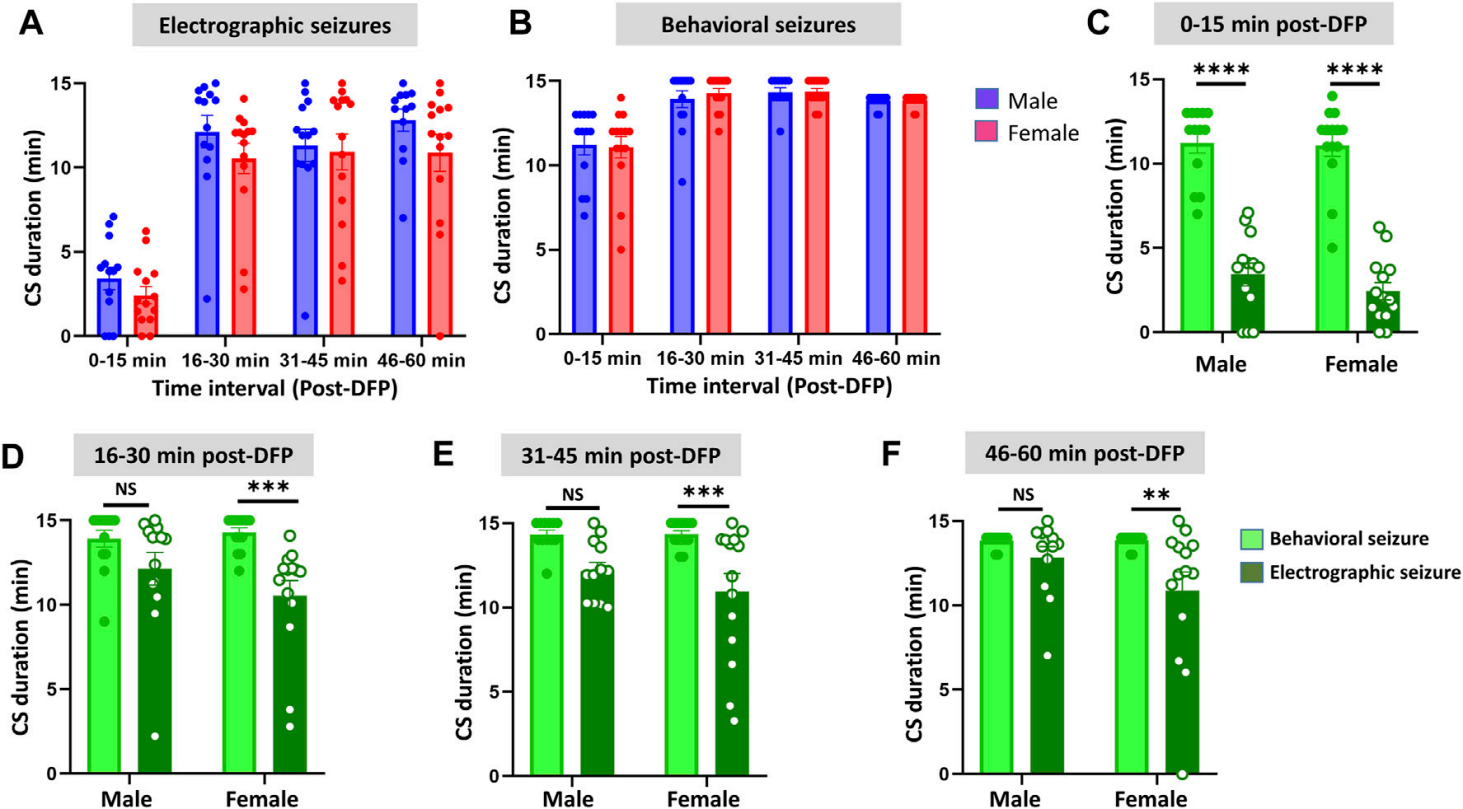
Time-segment wise electrographic seizures **(A)** and behavioral seizures **(B)** severity during 60 min post-DFP exposure. There were no sex differences at any time segments **(A,B)**. There were significant reductions in both sexes during the first 15 min **(C)** and at all time segments in females but not in males **(D–F)** Mixed-effects analysis [**(A,B)** *n* = 13–14], 2-way ANOVA [**(C,D)** *n* = 13–14; **(E,F)**, *n* = 12–14]. **p = 0.0042, ***p < 0.001, ****p < 0.0001. NS, non-significant.

**FIGURE 7:**
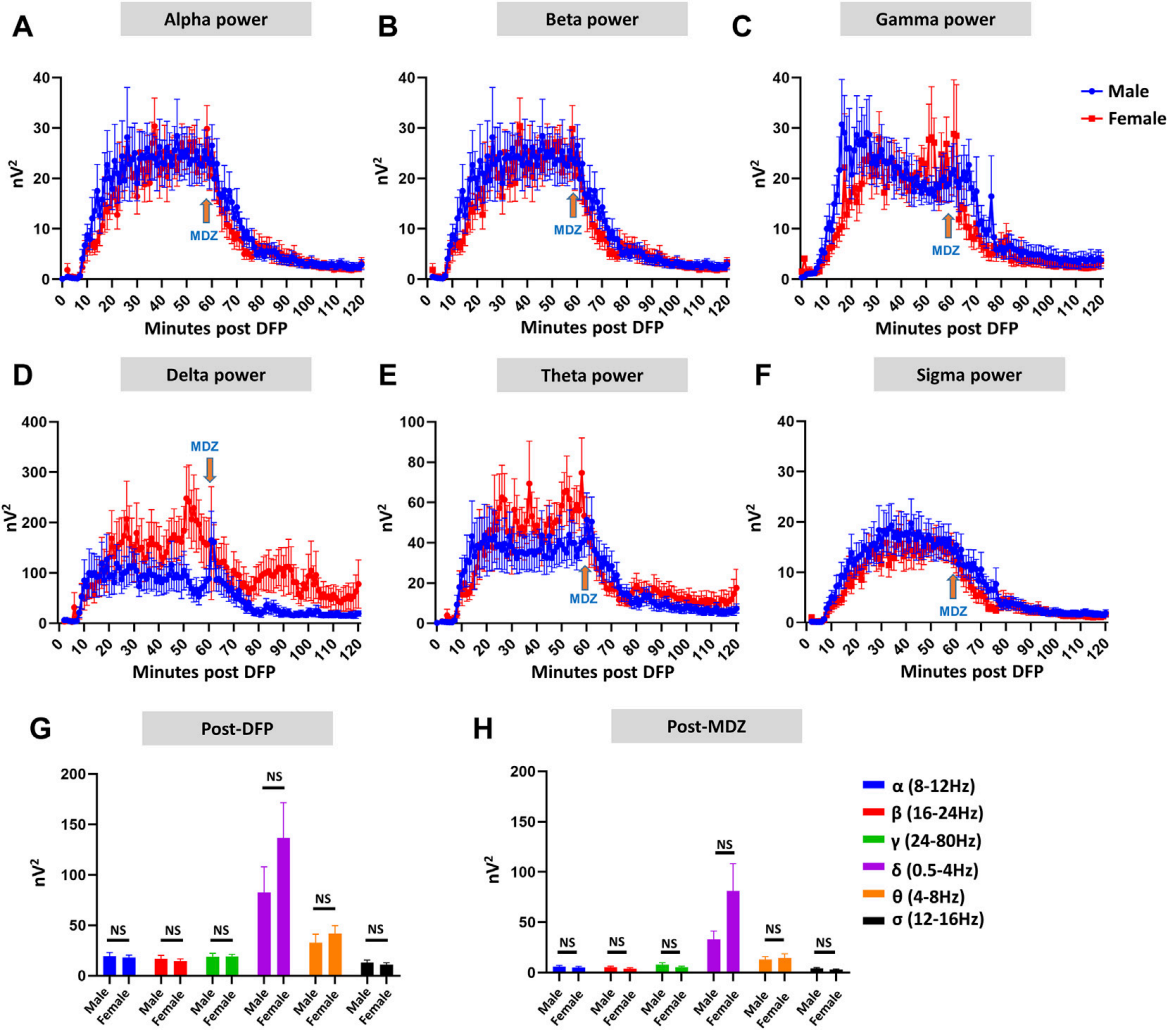
EEG power analysis overtime during the 120 minutes of post-DFP exposure and comparison between males and females **(A–H)**. Power-wise comparison between males and females did not show significant sex differences **(G,H)**. The delta and theta powers were higher in females at certain time points than in males **(D,E)**. Mann-Whitney test [**(F)** (*α*, *δ*, *θ*, *σ*), **(G)** (*α-σ*)], Unpaired *t*-test [**(F)** (*β*, *γ*)]. NS, non-significant.

**FIGURE 8:**
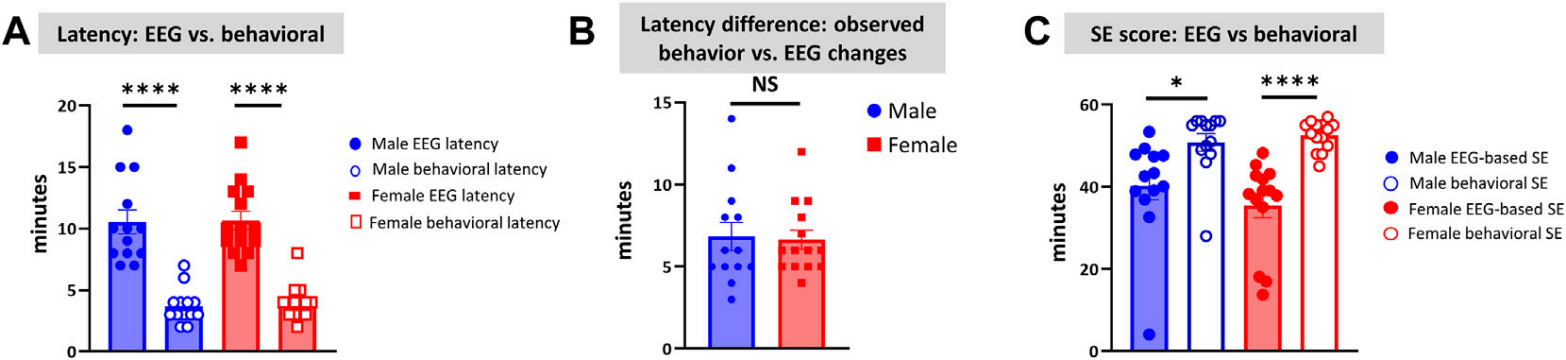
Latency differences between the observed behavioral CS and EEG changes were significant in both sexes **(A)**. Both male and female rats had similar latency in behavioral and electrographic seizures **(B)**. There were also significant differences in the overall duration of CS in both sexes between direct observation of behavioral CS and EEG-based CS quantification **(C)**. Mann-Whitney test [**(B)** *n* = 13–14]. *p = 0.0106, ****p < 0.0001. NS, non-significant.

Representative EEG traces, the corresponding behavior and the power spectra from a male and female rat, and the CS stage-specific spike characteristics are shown in Figure 4. As expected, the epileptiform spiking on EEG increased over time in males and females after exposure to DFP (Figure 5A). The latency to CS-associated spiking pattern on EEG was about 10 min in both males and females (Figures 5A, 8A). Once the animals reached a CS stage, the epileptiform spike rate and SE severity did not change significantly until they received MDZ (Figures 5A–C). In both sexes, MDZ significantly reduced the spike rate compared to the pre-MDZ period of the same duration (*p* < 0.0001; Figure 5B). There were no sex differences in their response to MDZ. There were also no significant differences in EEG-based SE severity between sexes before MDZ treatment (Figure 5C). We also further analyzed the average behavioral and electrographic seizure scores at different blocks of time (0–15 min; 16–30 min; 31–45 min, and 46–60 min) between DFP exposure and MDZ treatments (Figure 6). These analyses also confirmed no sex difference in SE severity in behavioral and EEG-based SE scores (Figures 6A,B). However, when behavioral SE scores were compared with EEG-based SE scores, there were significant differences in females at all time blocks, while in males, the differences were significant at 0–15 min only (Figures 6C–F). We further compared the power spectral differences between males and females over time. Interestingly, females appeared to have higher delta and theta powers than males. However, it was not statistically significant (Figures 7D,E,G). Overall, there were no significant sex differences for any power spectra (Figures 7A–H).

### Discrepancies Between the Observed Behavioral CS Versus EEG Changes and the Latencies

**FIGURE 9:**
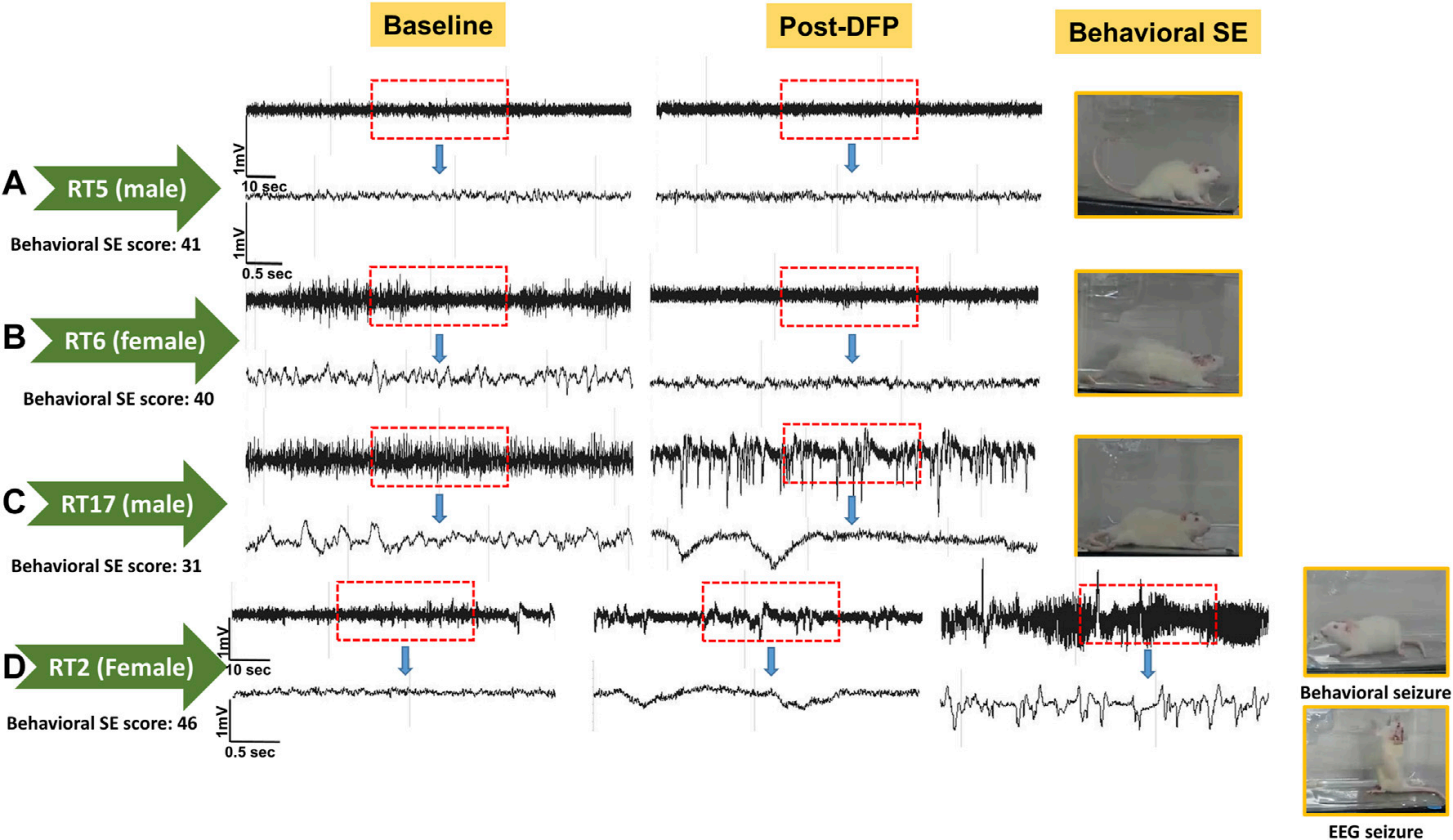
EEG traces from the two rats that did not show EEG changes during post-DFP exposure **(A,B)**. Basal EEG (pre-DFP) and post-DFP EEG traces are shown side-by-side for each animal, suggesting no signal interference or issues with electrodes. C, D traces are from the rats that had seizures ∼1 or 3 min before MDZ injection, i.e., about 57 or 59 min of latency.

Unlike the KA model of epilepsy, OP chemoconvulsants have both central and peripheral effects. Although the animals were treated with atropine sulfate and 2-PAM to counteract the peripheral effects of AChE inhibition, we wanted to verify whether the seizures originated in the brain. Therefore, the initial SE severity in telemetry rats was scored by direct observation and EEG-based approaches. In both sexes, there was a significant difference between the observed behavioral latency and the EEG-based latency to the onset of the first CS (*p* < 0.0001; Figure 8A). The first 5 min of the observed behavioral CS did not correlate with EEG-based CS patterns in both sexes (Figure 8B). There was also an occasional mismatch between behavioral and EEG changes after the first CS. Therefore, there were also significant differences in the duration of CS between observed behavioral and EEG-based quantification in both sexes (*p* = 0.01, *p* < 0.0001; Figure 8C). Overall, we found that all EEG-based CS patterns correlated with behavioral CS (confirmed by the integrated video) but not all behavioral CS (directly observed by experimenters) correlated with EEG changes, especially in the first 15 min of post-DFP (Figure 6C). Interestingly, two animals with ∼40 minof behavioral CS did not show any change on EEG post-DFP. Two other rats had about 3 min of EEG-based CS patterns before the MDZ injection. However, the baseline EEGs of all these rats were normal (Figure 9). Two more rats also had prolonged latency with occasional EEG seizures though they had severe behavioral SE as the other rats. A summary of the rats that were exceptional to the general trend, their behavioral SE severity, latency, and spike rate are compiled in Table 3. These animals were excluded from EEG analysis (Figures 5–8).

**TABLE 3:** The list of animals excluded from spike counts and electrographic seizure quantification. SE severity is the duration of convulsive seizures (CS, in minutes) ≥stage-3 between DFP and MDZ treatment.

Animal ID	Sex	Observed behavioral SE severity and latency (in min)	SPM post-DFP	EEG-based SE severity and latency (in min)	Comments
RT2	Female	46 (7)	5.30	3.1 (57)	Prolonged latency, occasional EEG seizures
RT3	Male	33 (4)	28.28	2.67 (19)	Prolonged latency, occasional EEG seizures
RT5	Male	41 (7)	0.12	0	No changes on EEG (but normal baseline)
RT6	Female	40 (5)	0	0	No changes on EEG (but normal baseline)
RT17	Male	31 (5)	16.21	0.78 (59)	Prolonged latency a single seizure
RT22	Female	57 (2)	136.08	6.15 (28)	Prolonged latency, occasional seizures

The numbers in parenthesis indicate the latency to the onset of CS. Representative EEG traces are shown in Figure 9. SPM, spikes per minute.

## Discussion

Our previous DFP studies were conducted in male and female rats at different time points, with or without telemetry devices (Gage et al., 2020, 2022b; Putra et al., 2020a, 2020b). The published literature on other chemoconvulsants such as KA and pilocarpine models (Tan and Tan, 2001; Scharfman and MacLusky, 2014; Smith et al., 2015), and our previous studies in the rat DFP model, led to the hypothesis that sex differences exist in SE severity in response to chemoconvulsant exposure. The animals were bought from the same vendor for all our experiments, including this study (Charles River, United States). The source of the DFP was from the same company (Sigma Aldrich, United States ) for all our experiments, but the batches were different. In our previous studies, female rats always received higher doses than males to achieve a similar SE severity (Gage et al., 2020; 2021a; 2021b; 2022a). In this study, we also used the DFP from different batches but the same vendor as our previous studies. We administered the DFP as a single dose of 4 mg/kg to both males and females at the same time from the same pool of reconstituted drugs. In this study, the male and female animals were used as a mixed-sex cohort, initially ignoring sex as a variable. Furthermore, unlike in our recent soman study (Gage et al., 2022b), we did not conduct vaginal cytology to determine the estrous cycle stages before DFP exposure to rule out the handling stress-induced variability. However, as we did in the soman study, we conducted experiments in both non-telemetry and telemetry rats in a mixed-sex cohort (housed in the same room). We compared the latency and severity of SE between groups to determine the impact of surgery and sex. Therefore, this study is a well-controlled rigorous experiment that addresses the Rigor and Reproducibility concept of the NIH, which has been a concern in experimental models of epilepsy.

Initial SE severity is an important variable in translational experimental models of epilepsy, especially the studies investigating the efficacy of a test drug versus a vehicle (Puttachary et al., 2016a; Putra et al., 2020a; Sharma et al., 2021). Achieving consistency in SE severity between batches and between sexes has been challenging. In our previous studies in the rat and mouse KA models, a modified approach, i.e., repeated low doses instead of a single high dose of KA, mitigated variability in initial SE severity (Tse et al., 2014; Puttachary et al., 2016a; Sharma et al., 2018a; Sharma et al., 2021). However, a repeated-low-dose approach is not suitable for DFP, soman, or other OPNA models as it does not mimic the real-world exposure of OPNAs to the general public. We and others have demonstrated that both DFP and soman models demonstrated the classical features of epilepsy, such as the onset of spontaneously recurring CS, gliosis, and neurodegeneration (Apland et al., 2014; Apland et al., 2018; Lumley et al., 2019; Putra et al., 2020a, 2020b; Rojas et al., 2020; Gage et al., 2021a, 2022a, 2022b; González et al., 2021; Reddy et al., 2021). Off note, some of these studies used pyridostigmine bromide (PB) or other medical countermeasures such as HI-6 or 2-PAM as a pretreatment to minimize mortality (Anderson et al., 1992; Apland et al., 2014; de Araujo Furtado et al., 2010; Reddy et al., 2020, 2021). Although some studies show that pretreatment has no impact on SE severity induced by DFP or soman, PB usage in a mixed-sex cohort can be a problem since females are less responsive to PB and confound SE severity and sex differences (Hernandez et al., 2020). The initial SE severity determines the onset of epileptogenesis in these models (Putra et al., 2020a; Gage et al., 2021a). The animals that had <20 min of SE in the rat DFP model did not develop epilepsy (Putra et al., 2020b). The initial SE severity also determines the extent of brain pathology in the rat DFP model. We defined SE severity as the duration of CS (stage ≥3) in minutes that an animal had between DFP and MDZ injections. Our previous study found that if animals had SE for ∼20 min, the pathology would be limited to the amygdala and piriform cortex. In severe SE, i.e., >30 min, the hippocampus and the other brain regions are affected in addition to the amygdala and piriform cortex (Gage et al., 2021a). Therefore, achieving relative consistency in SE severity, whether mild or severe, between sexes, is vital for interpreting the efficacy of disease-modifying test drugs in OPNA models. In one of our past DFP studies, since the initial SE severity was compromised in a batch of rats, we could not draw meaningful conclusions about the drug efficacy in females (Gage et al., 2020).

Sex is a critical biological variable in all animal models of epilepsy. Unless we control other variables, such as initial SE severity and age, the impact of sex on anticipated outcomes can be problematic to interpret the efficacy of an investigational drug. In the past decades, the vast majority of the investigational new drugs were experimented on male animals (Beery and Zucker, 2011). The NIH Health Revitalization Act issued guidelines to include females in clinical trials in 1993 (The NIH policy and Guidelines). Subsequently, the NIH mandated that sex as a biological variable (SABV) be factored into research designs, analyses, and reporting in vertebrate animal and human studies (Clayton and Collins, 2014). Importantly some drugs in humans did not show efficacy in females or had adverse effects (Franconi et al., 2007; Holdcroft, 2007; Tingen et al., 2010; Zucker and Prendergast, 2020). The reluctance to use females in experiments has been likely due to the effects of the estrous cycle stages on experimental outcomes (Yoon et al., 2014; Beery, 2018). The hormone levels fluctuate with the estrous cycle stages, similar to humans though the rat estrous cycle is shorter, i.e., 4–5 days (Staley and Scharfman, 2005; Plant et al., 2015). As hormone levels influence the biological processes, the estrous staging is factored in experimental design in models of epilepsy or seizures.

The inclusion of females in epilepsy models is compelling since ∼40% of females with epilepsy have catamenial epilepsy, likely due to a reduction in seizure threshold in response to hormonal changes (Penovich and Helmers, 2008; Herzog, 2015). Animal models of chemoconvulsants-induced epilepsy showed female resistance to the induction of seizures compared to males for the same dose of pilocarpine but not with KA (Scharfman and MacLusky, 2014). In the sarin model, female rats in proestrus required a higher concentration to induce SE than other females in estrus and males (Smith et al., 2015). There were sex-dependent differences in SE in a picrotoxin model due to hormones-induced changes (Tan and Tan, 2001). In our recent study in the soman model, female rats in non-telemetry, irrespective of the stage of the estrous cycle, had severe CS (stage ≥3) for 44 min, while males had an average of 32 min (Gage et al., 2022b). It is plausible that handling and procedural stress, induced during vaginal lavage for cytology, might have impacted females’ response to soman. Interestingly, there were no sex differences in SE severity in response to soman in telemetry devices implanted animals. In our previous study, telemetry-implanted females exposed to DFP had significantly less severe SE and decreased epileptiform spiking than males, although females received a higher dose (4 mg/kg) of DFP than males (3 mg/kg). The estrous stages had no impact on seizure susceptibility, but rats with severe SE had significantly prolonged diestrus (Gage et al., 2020). Interestingly, soman telemetry rats did not show any estrous-dependent differences. Based on these findings, in the present study, we did not determine the estrous cycle stages in both telemetry and non-telemetry experiments. As a mixed-sex cohort housed in the same room, we found that both males and females responded with similar SE severity to the same dose of DFP (4 mg/kg).

Continuous, integrated, video-EEG recording to detect seizures and epileptiform discharges have been a gold standard, unbiased technique in experimental models of epilepsy to determine the efficacy of disease modifiers. However, drilling holes through the skull to implant electrodes on the brain’s surface reduces the seizure threshold in response to chemoconvulsants (Sharma et al., 2018b). We recently demonstrated the impact of depth electrodes on altered proteome levels in the hippocampus and cytokine levels in the brain and serum (Tse et al., 2021). In all our recent studies, the electrodes were placed epidurally rather than in the brain. We also verified any physical trauma to the brain after the animals were euthanized. None of the rats had depth electrodes in this study. In the rat soman model, the SE severity in the telemetry males was significantly higher than in non-telemetry males, which was similar to the KA model of epilepsy (Sharma et al., 2021; Gage et al., 2022b). In females, there were no differences in SE severity in telemetry and non-telemetry rats in the soman model since it had reached the maximum in non-telemetry animals (Gage et al., 2022b).

This study did not reveal significant sex differences in SE severity in either telemetry or non-telemetry groups. There were no significant sex differences in latency to the electrographic seizures and EEG-based SE severity. There were also no significant sex differences in any power spectra. Both sexes responded to MDZ and significantly reduced epileptiform discharges, and there were no sex differences. Previous studies in the soman model demonstrated that midazolam was the most potent and rapidly acting drug (McDonough et al., 1999). We have recently shown in the rat DFP model that midazolam prevents mortality. In contrast, diazepam could not rescue animals from DFP-induced mortality (Gage et al., 2021a). Our previous studies did not test the sex difference in MDZ efficacy. The cytochrome P450 3A (CYP3A) enzyme metabolizes MDZ in the liver and gut to its active metabolites 1-hydroxy-midazolam and 4-hydroxymidazolam. Although women showed significantly greater hepatic CYP3A activity than men, there are no convincing and reproducible reports on whether these active metabolites were also higher in women than men. Most studies reported that no sex differences were reported in MDZ efficacy (for example, Chen et al., 2006; Hu and Zhao, 2010). In a healthy human volunteers’ study, women exhibited 11% higher mean weight-corrected total body midazolam clearance and 28% higher oral clearance than men. However, they reported minor sex differences in area under curve (AUC) values (Chen et al., 2006). Therefore, it is not surprising that we observed no sex differences in the efficacy of MDZ in this study too.

The latency differences between the observed behavioral CS and the onset of electrographic changes, and the overall difference in SE severity in both sexes are intriguing observations in this study. These discrepancies could be due to the limited coverage of single-channel recording from epidurally implanted bipotential electrodes in this study. However, such discrepancy patterns were not observed in the kainate model when the same DSI transmitter devices were used in rats (Sharma et al., 2021). This suggests that the mismatch between the behavioral CS and the corresponding changes on EEG in <15 min of post-DFP exposure could be due to the effects of DFP in peripheral organs. The differences between 15–60 min post-exposure could be likely due to manually quantified seizures in one-minute epochs. The other interesting finding is that in four rats, there was significantly prolonged latency to the onset of the first electrographic CS pattern, although the behavioral CS latency was <8 min (Table 3). These observations suggest that the peripheral effects of atropine sulfate and 2-PAM may not be effective in some animals or may have a prolonged delayed effect. It would be interesting to investigate further; 1) the AChE levels in the peripheral tissues, 2) differences in brain pathology between the animals that had no EEG changes (despite behavioral CS) and the animals in which the behavioral seizures correlated with the electrographic changes. As reported in Table 3, the two rats (RT5 and RT6) with no EEG changes (despite 40 min of behavioral CS) had normal baseline EEG, and we did observe the expected day-night changes on the EEG baseline. These animals had occasional epileptiform spikes but no electrographic CS patterns. Therefore, it is unlikely due to epidurally placed electrodes or any technical issues. Furthermore, all other rats that showed CS changes on EEG (post-DFP) had similar baseline changes (day-night cycle). In these animals, AChE levels and their activation state in the brain and peripheral tissues may reveal the reason for behavioral vs. lack of EEG changes.

## Conclusion

This study demonstrated that mixed-sex cohorts of males and females housed in the same room minimize variability in SE severity in response to DFP exposure. Injections from the same pool of reconstituted DFP in cold PBS minimize variability, although the DFP used in this study was from different batches but the same vendor. SE severity quantification (i.e., CS stages and duration) by direct observation, video, and EEG analyses yielded reproducible results when the experiments were conducted in mixed-sex cohorts. We found no sex differences in SE severity in either the telemetry or non-telemetry cohort. The female rats showed decreased latency and increased SE severity in the telemetry group compared to the non-telemetry group. Both sexes responded to MDZ and there was no sex difference. Out of 35 telemetry animals, 29 animals’ EEG-based CS patterns correlated with the behavioral CS. The odd six animals had excellent EEG signals and normal baselines as the other animals, but surprisingly, there were no EEG changes in response to DFP despite severe behavioral CS (Table 3; Figure 9). Like the other animals, these six animals were also treated with atropine sulfate and 2-PAM to prevent peripheral effects of AChE inhibition by DFP. Our future studies will investigate whether brain pathology of these six animals correlates with the rest of the telemetry rats, which had similar behavioral SE severity. One of the limitations of this study is the lack of mechanistic or therapeutic aspects. The extension of this study will explore both a disease-modifying effect of an investigational new drug and its mechanism.

## Data Availability

The raw data supporting the conclusion of this article will be made available by the authors, without undue reservation.
